# Dietary branched-chain amino acids get to the heart of H3K23Pr

**DOI:** 10.1172/JCI174953

**Published:** 2023-11-15

**Authors:** Christina Demetriadou, Daniel S. Kantner, Nathaniel W. Snyder

**Affiliations:** 1 Department of Cancer Biology and; 2 Abramson Family Cancer Research Institute, University of Pennsylvania, Philadelphia, Pennsylvania, USA.; 3 Aging + Cardiovascular Discovery Center, Department of Cardiovascular Sciences, Lewis Katz School of Medicine, Temple University, Philadelphia, Pennsylvania, USA.

## Abstract

Cardiac metabolism provides effects that extend beyond the transformation of energy for the heart to operate effectively. Some metabolites also function as signaling molecules and exert transcriptional changes. Heart failure is a progressive pathology in which these metabolite functions falter. In this issue of the *JCI*, Yang et al. describe a protective effect from a low–branched chain amino acid (BCAA) diet in a mouse model of heart failure. The findings implicate a propionylation mark on histone H3 lysine 23 (H3K23Pr), previously shown to be dependent on the BCAA isoleucine, in transcriptional control of the cardiac stress response. The result underscores the interplay between metabolism and histone acylation, highlighting targeted dietary and pharmacological intervention as a means to decelerate cardiac hypertrophy.

## Histone lysine acylations and branched-chain amino acid metabolism

In eukaryotic cells, the genome is elegantly packed into structured chromatin with DNA wrapped around histone octamers. Covalent posttranslational modifications (PTMs) on histone proteins serve as part of a mechanism for the regulation of DNA-based processes, including transcription, DNA replication, and repair (1). Lysine acylation is one of the most prevalent histone PTMs, with the acyl group donated from metabolites that are intermediate or end products of core metabolic pathways including the TCA cycle, amino acid catabolism, and fatty acid oxidation (2). As a result, shifts in metabolite concentration influence the occurrence and types of histone acylations, thereby altering the chromatin landscape and gene expression patterns.

Histone lysine propionylation (KPr) is a PTM that involves the addition of a three-carbon propionyl group from propionyl-CoA (prop-CoA) to the ε-amino group of a lysine side chain (3). Prop-CoA is well known in cardiac metabolism as an intermediate in one of the major anapleurotic pathways to the TCA cycle in the mitochondria, via the conversion to d-methylmalonyl-CoA, followed by racemization to l-methylmalonyl-CoA and isomerization to succinyl-CoA (4). Prop-CoA can be derived from propionate, odd-chain fatty acids, cholesterol, methionine, threonine, and the branched-chain amino acid (BCAA) isoleucine or valine. In other subcellular compartments, including the nucleus, prop-CoA can act alternatively as a donor for KPr. Previous work has demonstrated that a pool of prop-CoA exists in the nucleus and that this pool of prop-CoA can be preferentially derived from isoleucine and act as a donor for specific KPr sites, including histone H3 lysine 23 propionylation (H3K23Pr) (5). The function of H3K23Pr in any cardiac context remained unknown until this study by Yang et al. (6).

## BCAA intake and cardiac hypertrophy

**Figure 1:**
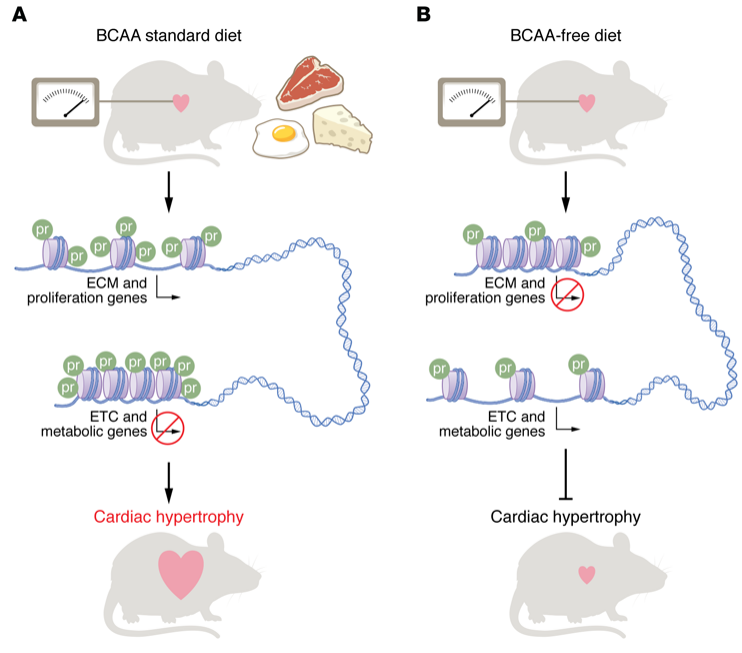
Yang et al. (6) demonstrate a cardioprotective role of a BCAA-free diet in a mouse model of heart failure. Hearts from mice fed a standard BCAA diet and subjected to short-term pressure overload exhibited enrichment of propionylation on H3K23Pr at the promoters of specific workload-dependent genes. Enrichment of H3K23Pr correlated with increased expression of ECM and proliferation genes, as well as reduced transcription of metabolic and ETC genes relative to unstressed hearts. The pressure overload–induced enrichment of H3K23Pr at those loci was prevented in mice fed a diet lacking BCAAs, which also restored gene expression changes and inhibited cardiac hypertrophy and failure. This study defines a role of H3K23Pr in the context of cardiac health.

In this issue of the *JCI*, Yang et al. asked whether dietary BCAA intake affects cardiac hypertrophy through modulation of KPr and transcription in the mouse heart. The authors initially demonstrated that feeding mice a low-BCAA diet impeded the development of work overload–induced cardiac hypertrophy (Figure 1). This finding aligns with a wider implication of BCAA metabolism dysregulation in heart failure that remains plagued by a few unresolved apparent contradictions (7). The heart itself does not seem to appreciably oxidize BCAAs, especially compared with the avid use of other substrates that ultimately drive oxidative phosphorylation and generate ATP. Thus, a question emerged: How, specifically, does food intake of BCAAs contribute to heart failure?

Given the previous work on localization of subunits of the branched-chain α-keto acid dehydrogenase (BCKDH) complex, the contribution of isoleucine-derived carbons to site-specific histone KPr, and the recent understanding of specific KPr marks in disease, Yang and authors sought to investigate whether BCAA metabolism controls cardiomyopathy through modulation of H3K23Pr. They found that hearts subjected to short-term pressure overload in mice maintained on a standard BCAA diet did not exhibit marked changes in the average levels of H3K23Pr across the genome (6). However, the hypertrophied hearts showed augmented H3K23Pr enrichment on the promoters of specific workload-dependent genes in comparison with the unstressed hearts. This locus-specific increase in H3K23Pr coincided with the upregulation of genes involved in extracellular matrix (ECM) such as collagen, fibronectin, and laminin, or in proliferation such as KI67 (Figure 1). Some caution in interpretation may be warranted, as only a limited number of H3K23Pr antibodies are available, and the specificity of CHIP-Seq is intrinsically tied to the specificity of the antibodies used (8). Of note, pressure overload–induced H3K23Pr and activation of ECM genes was prevented in mice fed a diet lacking BCAAs but could be rescued by supplementing the BCAA-free food with propionate, an alternative source for KPr. As a result of compromised KI67 expression and reduced cell numbers due to the low BCAA or isoleucine supply, the authors suggest that dietary BCAAs might support heart fibroblast growth under work overload to accelerate cardiac pathogenesis. It remains to be seen whether the observed variations in H3K23Pr and gene expression were directly prompted by pressure overload or whether they represented secondary changes due to alterations in fibroblast proliferation rates.

Finally, the authors revealed that H3K23Pr was conversely reduced on the promoters of certain metabolic and electron transfer complex (ETC) genes whose expression was decreased upon pressure overload in mice fed a standard BCAA diet, therefore leading to impaired heart mitochondrial respiratory capacity (Figure 1). Surprisingly, downregulation of these genes in response to work overload was dependent on the presence of BCAAs but was not affected by propionate supplementation. Hence, limiting BCAAs in the diet facilitated mitochondrial respiration in the diseased heart and impeded the progression of cardiac hypertrophy (6).

## Implications of modifying cardiac histones

Yang et al. (6) raise immediate questions for both translational and fundamental research. First, can a dose-response relationship be established for BCAA reduction that would translate to humans? The dose response between an acyl donor and the corresponding histone mark(s) and the resulting effects on transcription and cell behavior are likely to be complex. In patient populations, prolonged BCAA-free diets may be difficult to achieve. If the relevant substrate is, in fact, isoleucine only, can a dose-response relationship be established, and how is this specificity among the propiogenic substrates achieved? The metabolic basis of an isoleucine-restricted effect is not understood, so fundamental cellular biochemical experiments to elucidate this phenomenon are critical.

From a mechanistic standpoint, a second question arises: Can the same cardioprotective effect be reproduced without a BCAA reduction by targeting H3K23Pr? H3K23Pr is among the sites upregulated in a high-fat-diet mouse model (9), while BRPF1/KAT6-mediated histone acylation on lysine 23 has been implicated in neurodevelopmental disorders (10). More selective KAT6 inhibitors are being explored for treatments in other conditions (11). To our knowledge, no so-called “eraser” histone deacylases that remove Pr from H3K23 have been identified yet. Techniques, including systems-level approaches to quantify histone acylations (12) and screens including coupled CRISPR-ChIP may be useful in identifying the modifiers of such unexplored metabolite-responsive marks (13). In this particular case of cardioprotection, such approaches may reveal whether KPr is a general mark of active chromatin, since previous work showed that H3K14Pr stimulates gene expression (3), whereas H3K23Pr, as shown by Yang et al. in this work, may have repressive functions (6).

In summary, the cumulative data from this provocative work delineate a functional role of specific histone propionylation in cardiac physiology and strengthen the potential clinical usage of dietary interventions to cure work overload–induced heart failure (6). More broadly, these findings expand our understanding of the regulation of transcription by chromatin acylation in response to nutritional cues. This is an exciting advance for both translational and fundamental research in metabolite-epigenetics crosstalk.

## Version 1 11/15/2023

Electronic publication
